# Editorial of Conjugated Polymers: Preparation, Properties and Applications

**DOI:** 10.3390/polym17060710

**Published:** 2025-03-07

**Authors:** Paolo Coghi, Carmine Coluccini

**Affiliations:** 1 Laboratory for Drug Discovery from Natural Resources & Industrialization, School of Pharmacy, Macau University of Science and Technology, Macau 999078, China; 2 Institute of New Drug Development, College of Medicine, China Medical University, No. 91 Hsueh-Shih Road, Taichung 40402, Taiwan

Conjugated polymers (CPs) continue to revolutionize the landscape of materials science with their unique electrical, optical, and mechanical properties. Recent advancements in their synthesis, doping strategies, and structural modifications have opened new avenues in photovoltaics, optoelectronics, biomedical applications, and energy storage. The collection of recent studies highlights the remarkable versatility of CPs and their increasing significance in both fundamental research and practical applications (Figure 1).

One of the notable developments in CP research is the electropolymerization of inherently chiral polymer films, as demonstrated by Niebisch et al. [1]. This study showcases a novel diketopyrrolopyrrole-based polymer that exhibits chiroptical properties, expanding the potential for CPs in chiral sensing and optoelectronic applications. Meanwhile, the work of Yue et al. [2] emphasizes the critical role of dopant selection in fine-tuning the electrical transport properties of CPs. Their investigation into the effects of different chemical dopants on indacenodithiophene-co-benzothiadiazole polymers provides valuable insights into optimizing CPs for industrial-scale applications.

In the realm of nanoelectronics, Chen et al. [3] introduce high-performance one-dimensional transistors based on poly(p-phenylene ethynylene) molecular wires. Their findings indicate that gate-all-around molecular wire FETs could serve as a promising candidate to extend Moore’s law, bringing transistor technology closer to the sub-5 nm scale. Similarly, Lian et al. [4] explore the incorporation of benzimidazole structures into polyimides, significantly enhancing their thermal and dielectric properties for flexible electronic applications.

Biomedical applications of CPs have also seen remarkable advancements. Trindade [5] provides an extensive review of elastomeric Janus particles with controlled surface textures, highlighting their potential in bacterial adhesion studies and biomimetic materials. Krawczyk et al. [6] report the development of a polypyrrole-based drug delivery system tailored for neurological applications. Their study demonstrates the successful incorporation of chlorpromazine and heparin into CP matrices, paving the way for precise, controlled drug release mechanisms.

Photonic applications remain a major focus for CP research, as highlighted by Coghi et al. [7] in their review of light-sensitive conjugated polymers for photovoltaic and light-emitting devices. The ability of CPs to efficiently absorb and convert light energy is also demonstrated in the work of Zulkifli et al. [8], where a novel polycyclopentadithiophene-based polymer exhibits promising photocatalytic activity for organic transformations. Additionally, Nurazizah et al. [9] provide a systematic comparison of PEDOT:PSS and PEDOT:Carrageenan in dye-sensitized solar cells, revealing their respective roles as counter-electrodes and electrolytes, and suggesting pathways for improving their efficiency.

Advances in theoretical and computational chemistry have furthered our understanding of CP behavior at the molecular level. Rodríguez-Sánchez et al. [10] conduct a theoretical study on vinyl-sulfonate monomers and their impact as dopants in polyaniline dimers, shedding light on their electronic properties and reactivity. Lim et al. [11] provide a computational perspective on nitrogen-substituted polycyclic aromatic hydrocarbons, elucidating their π-electron delocalization patterns and potential electronic applications. Finally, Šloufová et al. [12] present a novel class of metallo-supramolecular polymers featuring 1-thioxophosphole units, revealing intriguing photoinduced Raman spectral changes that contribute to the growing field of responsive polymeric materials.

Taken together, these studies underscore the transformative potential of conjugated polymers across a broad spectrum of scientific and technological domains. As research continues to push the boundaries of CP design and application, it is evident that these materials will play an increasingly vital role in shaping the future of electronics, medicine, and sustainable energy solutions.

We extend our gratitude to all contributing authors and reviewers who have helped shape this Special Issue, and we look forward to further advancements in this rapidly evolving field.

## Figures and Tables

**Figure 1:**
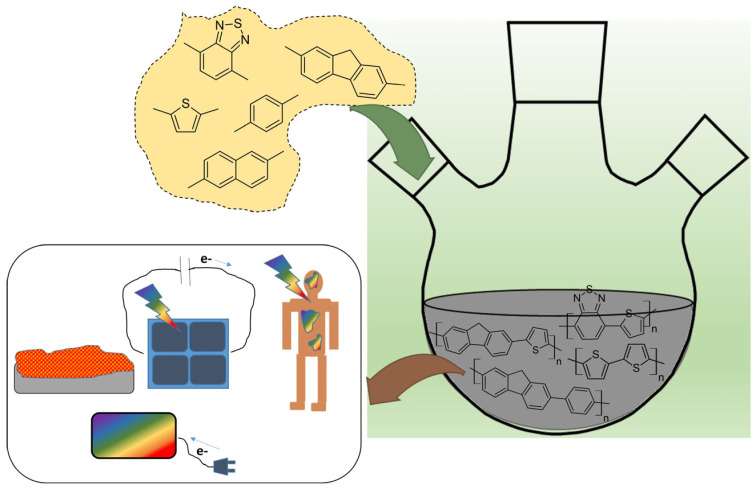
Schematic overview highlighting key aspects of synthesis strategies, fundamental properties, and diverse applications in electronics, optoelectronics (bioanalysis), and energy production (Organic photovoltaic).
